# First person – Angela M. Alicea-Serrano

**DOI:** 10.1242/bio.061953

**Published:** 2025-05-06

## Abstract

First Person is a series of interviews with the first authors of a selection of papers published in Biology Open, helping researchers promote themselves alongside their papers. Angela M. Alicea-Serrano is first author on ‘ Viscid silk in spider orb webs adheres strongly across surfaces with different roughness and surface energies’, published in BiO. Angela conducted the research described in this article while a PhD candidate in Todd A. Blackledge's lab at The University of Akron, Akron, USA. She is now a National Science Foundation postdoctoral research fellow in biology in the lab of Ali Dhinojwala and Jessica E. Garb at the School of Polymer Science and Polymer Engineering, The University of Akron and University of Massachusetts, Lowell, USA, investigating how form, function, and environment shape natural materials, with a focus on spider silk biomechanics and its role in prey capture.


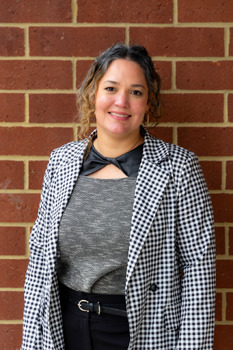


**Angela M. Alicea-Serrano**

**Describe your scientific journey and your current research focus**

Science has always fascinated me. Like many kids, I wanted to be a marine biologist, but in high school, chemistry was my favourite subject. In college, I majored in general sciences to explore different fields before committing to a career. That's when I discovered my passion for biology and research. I started my scientific career in a microbiology lab studying microbial ecology but soon realised I wanted to work outdoors. This led me to a spider evolution lab, where I searched for spiders in the dirt and studied their biology. After college, I pursued graduate school, researching spider silk material properties, combining both lab and fieldwork. Now, as a postdoc, I continue studying spider silk, from its molecular structure to its biomechanics. This integrated approach allows me to explore science from multiple angles, bridging fundamental biology with real-world applications.

**Who or what inspired you to become a scientist?**

My oldest sister was my first role model, she sparked my curiosity and guided me toward the natural sciences. Once I got to university, it was through inspiring professors and hands-on science classes that I truly discovered my passion for biology. Their enthusiasm and the excitement of exploring the natural world set me on the path to becoming a scientist.

**How would you explain the main finding of your paper?**

Our findings showed that the spider's glue can stick well to many different types of surfaces, from easily wetted surfaces to even those that repel water, like the lotus leaf. The glue is effective not only on smooth surfaces but can also stick to surfaces with unique features, like tiny bumps, which help the glue spread better. This ability to stick to a variety of surfaces likely limits the potential for insects to evolve anti-adhesive adaptations against spider webs.

By understanding how spider glue sticks to different surfaces, we could develop adhesives that work in tough environments

**What are the potential implications of this finding for your field of research?**

This finding could have big implications for our field. By understanding how spider glue sticks to different surfaces, we could develop adhesives that work in tough environments. This could be particularly useful in fields like medicine, where adhesives need to function on a range of surfaces, or in robotics and industry, where strong, reliable bonds are required in diverse conditions. In terms of biology, this finding suggest that insects have really limited potential to evolve changes in the surface energy or micromorphology of their cuticles to prevent sticking to spider webs. This really facilitates the ability of orb spiders to prey on a broad range of insects in their environments.

**Figure:**
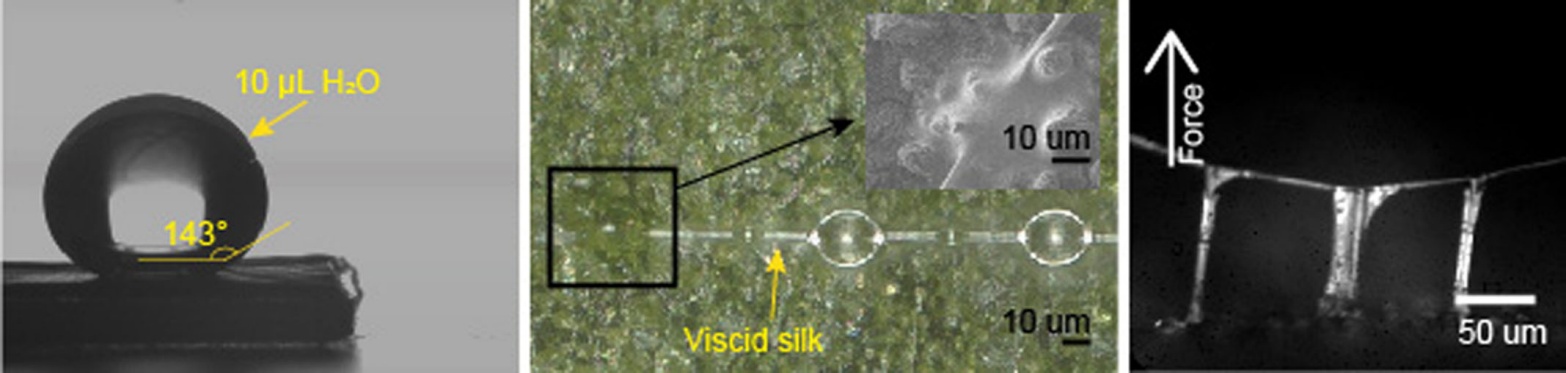
**Our study examines how water-based spider glues spread on superhydrophobic surfaces.** Our experiment demonstrates that spider glue droplets effectively spread on and adhere firmly to lotus leaves, one of the most hydrophobic surfaces in nature. The left image shows a lotus leaf, a surface where water does not spread. The middle image illustrates how spider glue on viscid silk spreads on the lotus leaf. The right image depicts the stickiness of the spider glue as the viscid silk is pulled from the lotus leaf.

**Which part of this research project was the most rewarding?**

The most rewarding part of this research was the collaboration between undergraduate and graduate students in designing the study and collecting data. It brought together researchers at different stages of their careers, creating a supportive and dynamic learning environment. Seeing new and experienced scientists work together, share ideas, and develop skills made the process not only productive but also a great learning experience for everyone involved.

**What do you enjoy most about being an early-career researcher?**

What I enjoy most about being an early-career researcher is the opportunity to come up with ideas and see them develop over time. Designing and carrying out experiments, watching the data come together, and ultimately shaping a scientific story is incredibly rewarding. The final step – sharing that story with the public – brings me the most joy, as it makes all the hard work feel meaningful and impactful.

**What piece of advice would you give to the next generation of researchers?**

My advice to the next generation of researchers is to get out there, explore, and try new things. It's normal to feel intimidated, but you won't know what excites you until you give it a shot. Science is all about curiosity and discovery, so embrace opportunities, take risks, and don't be afraid to step outside your comfort zone, you might find your passion in the most unexpected places.

**What's next for you?**

Next, I'm looking for a position where I can take on a leadership role as a lead scientist. I'm excited to not only contribute to research but also mentor the next generation of scientists, helping them grow and develop their skills just like I have.
